# Assessment of biodentine, mineral trioxide aggregate and enamel matrix derivative as direct pulp-capping agents

**DOI:** 10.6026/973206300212675

**Published:** 2025-08-31

**Authors:** Shivali Vaid, Ankitha Ade, Abhishek Mishra, Bonny Paul, Kavita Dube, Rishav Singh, Sanapala Mounika, Megha Ghosh

**Affiliations:** 1 Department of Oral Maxillofacial Pathology & Microbiology, MaharanaPratap College of Dentistry & Research Centre, Gwalior, Madhya Pradesh, India; 2 Department of Conservative Dentistry and Endodontics, Elite Dental Clinic and Maxillofacial Center, Ongole, India; 3 Department of Oral & Maxillofacial Surgery, ITS Dental College, Greater Noida, India; 4 Department of Conservative Dentistry and Endodontics, Hitkarini Dental College, Jabalpur, Madhya Pradesh, India; 5 Department of Pedodontics & Preventive Dentistry, Maharaja Ganga Singh Dental College & Research Centre, Sri Ganganagar, Rajasthan, India; 6 Intern, Kalinga Institute of Dental Science, Kalinga Institute of Industrial Technology (KIIT) Deemed to be University, Patia, Bhubaneswar, Odisha, India; 7 Department of Conservative Dentistry and Endodontics, College of Dental Sciences and Hospital, Rau, Indore, Madhya Pradesh, Indore, India

**Keywords:** Biodentine, direct pulp capping, enamel matrix derivative (EMD), histology, MTA, pulpal reaction

## Abstract

Conservative pulp treatments are used to maintain the tooth's vitality. Therefore, it is of interest to assess the clinical, radiographic and histologic responses of the pulp-dentin complex after direct capping with the MTA, Biodentine and Enamel Matrix Derivative (EMD) in human permanent teeth. A sterile rotary round bur was used to mechanically expose the pulps of 36 premolars that were advised for extraction. The teeth were then divided to one of three experimental groups: MTA, Biodentine and Enamel Matrix Derivative (EMD). The creation of the dentine bridge was evaluated using radiographs and histology. Clinically absence of signs symptoms was noted. MTA had the highest dentine bridge formation, followed by biodentine and EMD.

## Background:

Restoring the tooth's shape and function while preserve the pulp's vitality is the goal of restorative therapy. When a tooth's pulpal chamber is exposed to the oral environment, microbes invade the pulp-dentin complex, which can result into pulpal inflammation and pathology if treatment is not received. The pulpal inflammation appears to be restricted around the carious lesion, according to a number of histologic investigations. Therefore, it is advisable to preserve the remaining healthy pulp if the contaminated tissue is removed [1]. To preserve the vitality of pulp exposed as a result of trauma or iatrogenic errors, vital pulp therapy has been used. Inflammation in deep carious lesions is limited to the surface of pulp [2]. Vital pulp therapy done to preserve the tooth pulp by eliminating any tissue contamination brought on by bacterial infiltration and by encouraging the repair or replacement of the calcified tissue barrier [3]. To maintain the coronal and radicular pulp tissue in a viable state, conservative pulp treatments are thus carried out [4]. The term "regenerative endodontics" describes tissue engineering techniques used to replace lost or injured tissues, such as the cementum, dentin, or tooth pulp. By shielding the pulp from the harmful effects of bacterial products, vital pulp therapy reduces the amount of damage to the pulp tissue. For immature teeth with pulp exposure, vital pulp therapy appears to be a better course of action [5]. One method for maintaining a vital pulp and restoring its healthy state is direct pulp capping (DPC), which involves applying a pulp protection chemical to the exposed area, similar to a protective wound dressing [6]. Proper sealing ability, antimicrobial activity, biocompatibility, bioactivity that can stimulate and modulate tissue formation, resistance to moisture sensitivity, non-absorbability to tissue fluid, dimensional stability, good strength, radio-opacity and ease of manipulation are all characteristics of the ideal pulp capping material [7].

The newest and most promising materials that are now undergoing testing for potential widespread application in dentistry are calcium silicate materials (CSMs). It has many beneficial uses in this discipline, including restoring perforated tooth structure and root-end filling. The first generation of CSMs, mineral trioxide aggregate (MTA), has a solid track record of clinical performance [3]. For permanent teeth, a variety of pulp capping materials, including MTA, biodentine and Emdogain, were proposed and tested [8, 9]. Mineral trioxide aggregate (MTA) is an antibacterial, bioactive and biocompatible substance with a high sealing capacity and other desirable qualities. Long setting times, high material costs, discolouration and challenging handling problems are some of MTA's drawbacks [10]. An improved calcium silicate-based substance makes up biodentine. It is brand-new restorative cement made up of calcium silicate. It doesn't discolour the tooth and has great biocompatibility, enhanced mechanical qualities and outstanding bioactive behaviour [11]. The hydrophobic enamel matrix proteins that make up enamel matrix derivative (EMD, Emdogain) are extracted from 6-month-old pig tooth buds that contain amelogenin, enamelin, tuftelin, amelin and ameloblastin [9]. Emdogain is a physiologically active pulp capping agent that promotes the development of mesenchymal cells. It promotes the production of hard tissue and causes the dental pulp to heal [1]. Therefore, it is of interest to describecurrent research to assess the clinical, radiograhic and histologic responses of the pulp-dentin complex after direct capping with the MTA, Biodentine and Enamel Matrix Derivative (EMD) in human permanent teeth.

## Materials and Methods:

After receiving ethical approval from the institutional ethics committee, this ex vivo study was carried out. The subjects gave their informed permission. The study's inclusion criteria were that only fully grown permanent premolar teeth that were recommended for extraction for orthodontic purposes, had no pathologic alterations on periapical radiographs and showed no symptoms of irreversible pulpitis. Trained single investigator carried out a standardised operative technique.

## Clinical procedure:

The pulps of 36 premolar teeth were physically exposed using a sterile rotary round bur following local anaesthesia and rubber dam isolation. After the exposure, a sterile cotton pellet soaked in saline solution was used to achieve haemostasis. Group I consisted of mineral trioxide aggregate (MTA-(ProRooT MTA, Dentsply, Germany), Group II of biodentine (Septodont Biodentine, France) and Group III of enamel matrix derivative (EMD-Emdogain-BIORA AB, Malmo, Sweden). These teeth were then assigned to one of the three experimental groups, each of which had twelve samples. The manufacturer's instructions were followed for mixing and applying the materials. After applying one of the pulp capping materials to the exposed pulp, the final restorative method (coronal restoration) involved applying a base of glass ionomer cement (GIC) (GC Fuji, Japan) and then a composite restoration. Information concerning the existence or lack of postoperative sensitivity was recorded. After that, the teeth underwent a three-month clinical and radiological evaluation. In contrast to the radiographic examination, which looked for PDL space widening, calcified barrier at the restored site and periapical radiolucency as seen on the IOPA, the clinical examination of the tooth in question included the presence or absence of postoperative pain, tenderness on percussion and neural sensitivity to cold test.

## Histologic evaluation:

Three months after clinical procedure, the teeth were extracted, decalcified for three to four days in 10% nitric acid, prepared using standard histologic procedures and embedded in paraffin wax. A microtome was used to cut pieces that were six micrometres thick and aligned with the tooth's primary vertical axis. After mounting the sections on glass slides, hematoxylin-eosin staining was applied. The modified criteria established by Faraco *et al.* [12] were used to perform the histomorphologic evaluations. According to the modified criteria, the degree of pulp inflammation (type, intensity and extension) and hard tissue formation (continuity, morphology and thickness) at the capping material interface were determined and scored on a scale of 1 to 4, where 1 denoted the most desired outcome and 4 the least desired. The midmost, thickest and thinnest point regions of the continuous dentinal bridge were used to measure the bridge's thickness. The three values' mean was determined. The collected data was statistically evaluated using SPSS software version 24.0 with ANOVA test, Shapiro-Wilk test and chi-square test, at p-value of less than 0.05.

## Results:

Seven patients (2 with biodentine, 4 with EMD and 1 with MTA) complained of spontaneous discomfort, within 2 weeks and they had root canal treatment. Other patients were responsive to electric and cold tests and did not exhibit any symptoms during the study period (Table 1 - see PDF). None of the teeth experienced periapical pathology at the 3-month follow-up. Definitive Dentin Bridge was formed 10 with MTA, 8 with biodentone and 7 with EMD group (Table 2 - see PDF). Table 3 (see PDF) indicates that pulpal response and calcific bridge formation was better with MTA followed by group II and III. Table 4 (see PDF) indicates that, biodentine had average calcified bridge formation with moderate pulpal inflammation whereas; EMD had highest pulpal inflammation and lowest calcified bridge formation suggesting that MTA and biodentine are good pulp capping materials.

## Discussion:

Reducing or eliminating pulp inflammation is one of the main objectives of pulp cap treatment. Reduced pulp inflammation could be a sign of improved pulp-capping material biocompatibility [8]. Using an agent with strong biocompatibility at the pulp exposure site is part of DPC [3]. According to our findings, MTA was superior in dentine bridge creation and reduced pulpal inflammation when used to correct purposeful pulp defects together with biodentine and EMD. When none of the following symptoms or indicators was observed, the treatment was considered successful: Internal and exterior root resorption, furcation radiolucency, swelling, fistulation, pathological movement, spontaneous discomfort, soreness on percussion, or periodontal ligament space widening. However, long-term success and eventual healing depend on the creation of a dent in bridge [2]. A clinical trial by Baume and Holz (1981) found that the likelihood of a satisfactory treatment outcome is lower for an infected pulp [13]. The current study's findings supported Katge and Patil's observations [14] by demonstrating that pulp necrosis was not seen. In carious teeth, Hegde *et al.* assessed the pulp-dentin complex's clinical response following DPC using MTA and biodentine. They came to the conclusion that when pulpal diagnosis is limited to reversible pulpitis, MTA and biodentine may be utilised as DPC agents [2]. The results are consistent with our findings. The main components of biodentine are calcium and silicon ions, which may have strengthened cell division and proliferation [15].

Zanini *et al.* evaluated biodentine in cell culture [16]. This study evaluated the potential of these novel materials in biomineralisation and odontoblast-like cell differentiation. The histomorphologic response of human dental pulps to direct pulp capping with MTA and Biodentine was assessed by Agrawal *et al.* that came to the conclusion that Biodentine was a viable substitute for MTA and an effective pulp capping agent [4]. Devi *et al.* used histologic analysis to evaluate the pulpal response of mineral trioxide aggregate (MTA), enamel matrix derivative (Emdogain) and biodentine as direct pulp capping (DPC) agents. In contrast to Emdogain, they found that pulp tissue tolerates MTA and Biodentine as DPC agents well [1]. This is in accordance to our results. Hoseinifar *et al.* examined how the human tooth pulp responded to DPC using biodentine, mineral trioxide aggregate (MTA) cement and calcium-enriched mixture (CEM). Six weeks following the intervention, the treated teeth were extracted and histopathologically assessed. In terms of dentine bridge production, they discovered no differences between MTA, CEM and biodentine; nevertheless, the thickness of the dentine bridge was greater in biodentine than in the other groups [8]. According to Youssef *et al.* MTA, Biodentine and Emdogain have comparable qualities and might be a superior pulp capping agent than Ca(OH)2. Every studied substance had an impact on dental pulp cells' ability to survive and encouraged in vitro pulp repair processes [17]. Karkehabadi *et al.* evaluated how the vitality of human dental pulp stem cells was affected by mineral trioxide aggregate (MTA), calcium enriched mixture (CEM) cement and biodentine with or without emdogain (EMD). They came to the conclusion that adding EMD to MTA and CEM cement could improve HDPSC viability after seven days [5]. In comparison to biodentine and EMD, we discovered that MTA had a superior pulpal response.

According to Eshghi *et al.* biodentine and MTA have comparable qualities and both materials exhibit high rates of clinical and radiographic success in primary tooth pulpotomy follow-up over an extended period of time [18]. Mahapatra *et al.* came to the conclusion that TheraCal LC, a resin-based calcium silicate agent, demonstrated good efficacy and may be utilised in practice with a good success rate in both clinical and radiographic settings as a capping agent. Abuhashema *et al.* evaluated the radiographic regenerative dentin development and clinical efficacy of mineral trioxide aggregate (MTA) and platelet rich fibrin (PRF) as direct pulp capping agents. They came to the conclusion that the clinical and radiographic success rate of direct pulp capping with Platelet Rich Fibrin (PRF) was similar to that of MTA [1]. According to Ayuob *et al.* Bio MTA plus is a great option for procedures meant to preserve or regenerate dental pulp tissue since it provides notable benefits in terms of biocompatibility, bioactivity and regenerative potential [20]. According to Boopathi *et al.* biodentine was more biocompatible than alendronate. Alendronate, however, has the ability to start the process of dentin repair [21]. According to a histologic research by Bollu *et al.* the combination of MTA and MTA/EMD resulted in a higher quality hard tissue response than using EMD alone. Regarding the development of calcified bridges and the inflammatory response of the pulp, there was no discernible difference between MTA/EMD and MTA [22]. To validate the findings on a larger sample size, more research is required.

## Conclusion:

MTA was found to be more effective than other examined groups in forming dentine bridges in pulp capping.
